# A comparative study of DA-9601 and misoprostol for prevention of NSAID-associated gastroduodenal injury in patients undergoing chronic NSAID treatment

**DOI:** 10.1007/s12272-014-0408-3

**Published:** 2014-05-30

**Authors:** Oh Young Lee, Dae-Hwan Kang, Dong Ho Lee, Il-Kwun Chung, Jae-Young Jang, Jin-Il Kim, Jin-Woong Cho, Jong-Sun Rew, Kang-Moon Lee, Kyoung Oh Kim, Myung-Gyu Choi, Sang-Woo Lee, Soo-Teik Lee, Tae-Oh Kim, Yong-Woon Shin, Sang-Yong Seol

**Affiliations:** 1 Department of Internal Medicine, Hanyang University College of Medicine, Seoul, South Korea; 2 Department of Internal Medicine, Pusan National University Yangsan Hospital, Busan, South Korea; 3 Department of Internal Medicine, Seoul National University Hospital of Bundang, Seongnam, South Korea; 4 Department of Internal Medicine, Soonchonhyang University Hospital Cheonan, Cheonan, South Korea; 5 Department of Internal Medicine, Kyung Hee University College of Medicine, Seoul, South Korea; 6 Department of Internal Medicine, The Catholic University of Korea Yeouido St.Mary’s Hospital, Seoul, South Korea; 7 Department of Internal Medicine, Presbyterian Medical Center, Jeonju, South Korea; 8 Department of Internal Medicine, Chonnam National University College of Medicine, Gwangju, South Korea; 9 Department of Internal Medicine, The Catholic University of Korea St.Vincent’s Hospital, Suwon, South Korea; 10 Department of Internal Medicine, Gacheon University Gil Medical Center, Incheon, South Korea; 11 Department of Internal Medicine, The Catholic University of Korea Seoul St.Mary’s Hospital, Seoul, South Korea; 12 Department of Internal Medicine, Korea University Ansan Hospital, Ansan, South Korea; 13 Department of Internal Medicine, Chonbuk National University Hospital, Jeonju, South Korea; 14 Department of Internal Medicine, Inje University Haeundae Paik Hospital, Busan, South Korea; 15 Department of Internal Medicine, Inha University Hospital, Incheon, South Korea; 16 Department of Internal Medicine, Inje University Busan Paik Hospital, 75 Bokji-ro, Busanjin-gu, Busan, 614-735 Korea

**Keywords:** DA-9601(Stilen^®^), Misoprostol, NSAID-associated, Gastroduodenal injury

## Abstract

Misoprostol is reported to prevent non-steroidal anti-inflammatory drug (NSAID)-associated gastroduodenal complications. There is, however, limited information regarding the efficacy of DA-9601 in this context. We performed a comparative study on the relative efficacy of DA-9601 and misoprostol for prevention of NSAID-associated complications. In this multicenter, double-blinded, active-controlled, stratified randomized, parallel group, non-inferiority trial, 520 patients who were to be treated with an NSAID (aceclofenac, 100 mg, twice daily) over a 4-week period were randomly assigned to groups for coincidental treatment with DA-9601 (60 mg, thrice daily) (236 patients for full analysis) or misoprostol (200 μg, thrice daily) (242 patients for full analysis). A total of 236 patients received DA-9601 and 242 received misoprostol. The primary endpoint was the gastric protection rate, and secondary endpoints were the duodenal protection rate and ulcer incidence rate. Endpoints were assessed by endoscopy after the 4-week treatment period. Drug-related adverse effects, including gastrointestinal (GI) symptoms, were also compared. At week 4, the gastric protection rates with DA-9601 and misoprostol were 81.4 % (192/236) and 89.3 % (216/242), respectively. The difference between the groups was −14.2 %, indicating non-inferiority of DA-9601 to misoprostol. Adverse event rates were not different between the two groups; however, the total scores for GI symptoms before and after administration were significantly lower in the DA-9601 group than in the misoprostol group (−0.2 ± 2.8 vs 1.2 ± 3.2; *p* < 0.0001). DA-9601 is as effective as misoprostol in preventing NSAID-associated gastroduodenal complications, and has a superior adverse GI effect profile.

## Introduction

Despite the various gastroduodenal complications associated with non-steroidal anti-inflammatory drugs (NSAIDs), they remain a desirable treatment for patients with musculoskeletal pain or other inflammatory conditions. NSAID-associated gastroduodenal complications include mild forms of gastropathy such as erosive gastritis and dyspepsia, in addition to more serious gastric or duodenal ulcers. Moreover, life-threatening gastrointestinal (GI) bleeding may be caused by chronic NSAID use (García Rodríguez and Jick 1994). These NSAID-associated gastroduodenal complications are attributable to the depletion of prostaglandin in the gastroduodenal mucosa, by inhibition of cyclooxygenase (COX), which is known to play an essential role in maintaining mucosal integrity (Schoen and Vender 1989). Replenishment of prostaglandin with misoprostol has, therefore, been used to prevent NSAID-associated gastroduodenal complications (Silverstein et al. 1995). In comparison with a placebo, misoprostol was shown to significantly reduce the incidence of gastroduodenal ulcers in chronic NSAID users (Graham et al. 2002; Hawkey et al. 1998).

Because NSAID-associated gastroduodenal injury can be facilitated by low intragastric pH, antisecretory agents such as H_2_ receptor antagonists (H_2_RAs) and proton pump inhibitors (PPIs) are commonly prescribed to reduce the risk of NSAID-associated gastroduodenal injury in patients being treated with NSAIDs (Agrawal et al. 2000). Administration of antisecretory agents is recommended for chronic NSAID users (Weaver and Gitlin 2002). When compared with PPIs, misoprostol has been demonstrated to be efficacious in preventing NSAID-associated gastroduodenal complications. Administration of misoprostol is associated with side effects including diarrhea, nausea, and abdominal pain. Studies comparing misoprostol with a H_2_RA or PPI have reported lower compliance in the case of misoprostol, preventing accurate assessment of drug efficacy.

In addition to COX inhibition and resultant prostaglandin depletion, NSAIDs may cause leukocyte recruitment and generation of reactive oxygen species (ROS), which may contribute towards gastroduodenal injury (Hussain et al. 2012). Inhibiting the development of ROS using antioxidants, such as DA-9601, may therefore represent a different modality for prevention of NSAID-associated GI complications. DA-9601 (Stillen) is a phytopharmaceutical derived from *Artemisia asiatica*, which has antioxidative and anti-inflammatory actions in the context of experimentally induced GI mucosal damage (Kim et al. 2004). In former double-blinded cetraxate-controlled phase III clinical studies, administration of DA-9601 was effective for treatment of erosive gastritis of various etiologies, and was well tolerated.

In a previous report, the preventive effect of DA-9601 and misoprostol on NSAID-associated gastroduodenal injury was established in healthy volunteers using a randomized, double-blinded, multicenter study. DA-9601 was shown to not be inferior to misoprostol in terms of endoscopic gastroduodenal protection rates. The study also indicated that the side-effect profile of DA-9601 was superior to that of misoprostol (Lee et al. 2011). However, compared with healthy volunteers, patients with arthritis are more susceptible to developing NSAID-associated gastropathies (McCafferty et al. 1995; Kato and Takeuchi 2002). Furthermore, the risk of development of a serious NSAID-associated peptic ulcer is increased in the elderly (Griffin et al. 1988, 1991; Hong et al. 2013).

We, therefore, conducted a multicenter, double-blinded, stratified randomized, active-controlled, parallel group, non-inferiority study to investigate whether DA-9601 is comparable to misoprostol in reducing NSAID-associated gastroduodenal complications. Both drug efficacy and safety were assessed.

## Materials and methods

### Study participants

Eligible patients were 20–65 years of age, on a course of NSAIDs for more than 4 weeks, experiencing pain due to rheumatoid arthritis, ankylosing spondylitis, osteoarthritis, frozen shoulders, toothache, trauma-associated inflammation, lumbar pain, ischialgia, or nonarthritic rheumatism, and had normal baseline endoscopic findings with no erosion or ulcer (i.e., grading scores of 0–1 based on gastric mucosal lesions, irrespectively of duodenal mucosal lesions) (Table 1).

**Table 1:** Endoscopic scoring of gastric mucosal lesions

Score	Gastric mucosal lesion status
0	No visible lesions
1	Mucosal hemorrhages only
2	1 or 2 erosions
3	Numerous (3–10) areas of erosions
4	Large number (>10) of erosions
5	Ulcer

Exclusion criteria were a history of gastric or duodenal ulcer within the previous 30 days, previous GI surgery, a history of hypersensitivity to any drug, drug dependency, any use of histamine 2 receptor antagonists, PPIs, sucralfate, misoprostol, DA-9601, triamterene, corticosteroids, cyclophosphamide, methotrexate, or NSAIDs (except for acetaminophen or aspirin not exceeding 200 mg/d) within 7 days of randomization. Pregnant or lactating females, and females of childbearing age not using contraception, were excluded. Patients were also excluded if they had major hematologic, renal, cardiac, pulmonary, and hepatic abnormalities, thrombotic disorders, consumption coagulopathy, acute hepatitis, pancreatitis, inflammatory bowel disease, or psychiatric disorders. Those with hypersensitivity to prostaglandin, aceclofenac, or drugs of the same class as aceclofenac, were also excluded.

### Study design

This study was a multicenter, double-blinded, stratified randomized, active-controlled, parallel group, non-inferiority trial conducted in South Korea from September 2012 to March 2014. Patients meeting the study criteria underwent a screening endoscopy within 1 week of the screening period, or, for those already taking an NSAID, after a run-in period of at least 1 week. During the washout period of 1 week, acetaminophen (Tylenol) was permitted as a rescue drug. Following the 1-week screening period, eligible patients were randomly assigned to either the DA-9601 (60 mg; Stillen; Dong-A ST Co., Seoul, Korea) or misoprostol (100 µg; Cytotec; Pfizer Pharmaceuticals Korea) treatment groups. DA-9601 and misoprostol were taken three times daily for 4 weeks, concurrent with twice-daily treatment with an NSAID (aceclofenac, 100 mg). Treatment assignments were carried out by a computer-generated randomization program that was designed to allocate patients to the two treatment groups in a 1:1 ratio. The subjects were assigned sequential allocation numbers at each site, and the medications were presented as two identical-appearing tablets containing an active drug or a placebo, in order to maintain the double blind condition. The subjects visited each center for follow-up endoscopy at week 4.

### Study assessments

#### Efficacy

Each subject underwent an upper GI endoscopy to establish a baseline, and again after the 4-week treatment period. The primary endpoint was the gastric mucosa protection rate at week 4, based on endoscopy scores ranging from 0 to 5 (0, no visible lesion; 1, mucosal hemorrhage only; 2, one or two erosions; 3, three to ten erosions; 4, more than 10 erosions; 5, ulcer) (Table 1). The protection rate was the percentage of patients who were shown to be protected at follow-up endoscopy (defined as endoscopy scores of 0–1) after 4 weeks of treatment. Secondary endpoints were the duodenal mucosa protection rate and the ulcer incidence rate at week 4. Drug compliance was assessed every day using a telephone-based interactive voice response system to ask patients whether they had taken the prescribed medications.

#### Safety

The incidence of adverse events, including any GI symptoms, and abnormalities in laboratory findings and vital signs, was assessed. GI symptoms consisted of abdominal pain, diarrhea, heartburn, nausea, vomiting, bloating, anorexia, and constipation.

### Ethics statement

The study was approved by the Institutional Review Board of each of the 36 participating institutions, including Inje University Busan Paik Hospital (IRB approval number: 11-183), The Catholic University of Korea Seoul St. Mary’s Hospital (KC11MSMV0970), Seoul National University Hospital of Bundang (B-1206-160-001), Severance Hospital Yonsei University (4-2012-0324), Wonkwang University Hospital (1368), Chonnam National University Hospital (CNUH-2012-004), The Catholic University of Korea St.Vincent’s Hospital (VC12MSMV0100), KyungHee University Medical Center (1217-04), Korea University Anam Hospital (AN12069-001), Korea University Ansan Hospital (AS12049), Kosin University Gospel Hospital (07), Daegu Catholic University Medical Center (CR-12-070), Pusan National University Hospital (H-1205-005-006), Kyungpook National University Hospital (KNUH 2013-01-036), Soonchunhyang University Hospital Cheonan (13), Chonbuk National University Hospital (CUH 2011-12-008-002), Chung-Ang University Hospital (C2012072(767)), Samsung Medical Center (SMC 2012-05-074), Kyungpook National University Hospital (KNUMC_12-0009), Ajou University Medical Center (AJIRB-MED-CT4-12-149), Gangnam Severance Hospital Yonsei University (15), Youngnam University Medical Center (YUH-12-0383-M26), Inha University Hospital (12-104), Hanyang University Medical Center (HYUH 2011-11-005), Hanyang University Guri Hospital (2012-029), Gachon University Gil medical Center (GBIRB2013-246), The Catholic University of Korea Yeouido St. Mary’s Hospital (SC13MSMV0158), Kangbuk Samsung Medical Center (KBC13168), Gyeongsang National University Hospital (GNUH 2013-09-013), Keimyung University Dongsan Medical Center (2013-08-004), Pusan Nsational University Yangsan Hospital (02-2013-023), Wonju Severance Christian Hospital (CR113029-001), Inje University Haeundae Paik Hospital (129792-2013-074), Presbyterian Medical Center (2013-08-31), Jeju National University Hospital (JEJUNUH 2013-07-014) and Dongguk University Ilsan Hospital (2013-115). The study was performed in compliance with good clinical practices, according to ICH guidelines.

### Statistical analysis

Based on the results of the previous study, the gastric mucosa protection rate, as assessed by endoscopy, was set at 86 and 95 % for DA-9601 and misoprostol, respectively. The non-inferiority margin was defined as 17 %, identical to that of the previous study (Lee et al. 2011). The number of subjects was determined assuming a level of one-sided significance, α, of 0.025 and an 80 % statistical power. The target sample size was computed as 420 patients in total; 210 patients per group. Considering an average dropout rate of 10 %, we aimed to recruit a total of 468 patients.

Demographic and baseline characteristics were summarized descriptively for all participating patient subjects. For inter-group differences, Student’s *t* test was used to evaluate the statistical significances of continuous data, and a Chi squared test was used for categorical data.

All efficacy analyses were performed on the full analysis set (FAS) and per-protocol set (PPS). The FAS population included all randomized subjects who received at least one dose of study drug and had at least one valid post-baseline efficacy evaluation. The PPS population was defined as a subset of the FAS population who completed the study without any major protocol violations.

For the primary efficacy analysis, a one-sided 97.5 % lower limit of difference rate between the two groups was computed. The gastric mucosa protection rate of DA-9601 (test group) could be considered non-inferior to misoprostol (control group) if the one-sided 97.5 % lower limit was greater than −17, the non-inferiority margin.

The differences in the duodenal mucosa protection rate and the ulcer incidence rate between the two groups were analyzed using Fisher’s exact test.

For adverse events, the number of patients who experienced one or more adverse drug reactions was recorded. Data are presented as percentages and two-sided 95 % confidence intervals (CI). Inter-group comparisons were conducted using a Chi squared test.

## Results

### Baseline characteristics

**Fig. 1:**
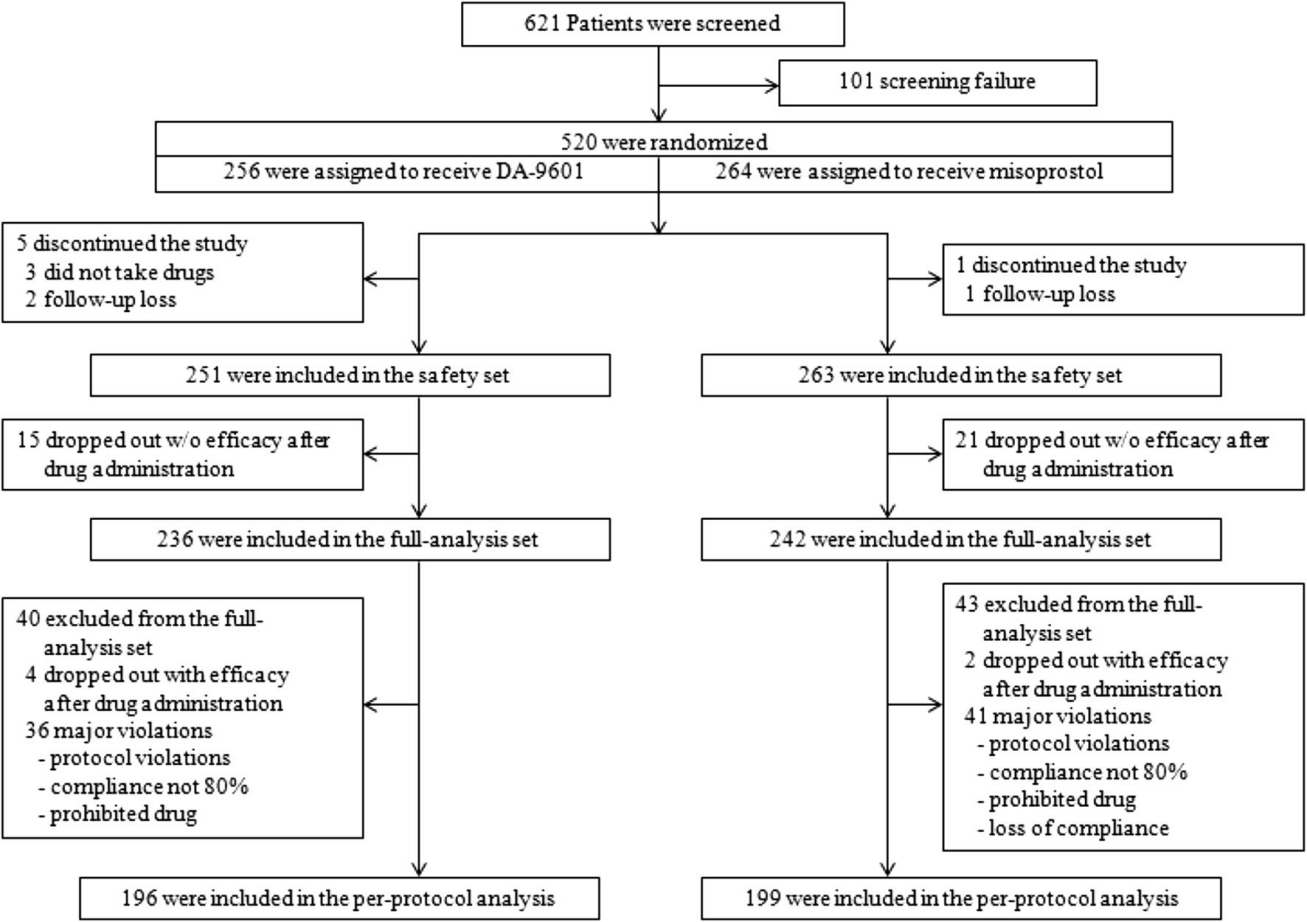
Enrolment, randomization, and follow-up

A total of 621 patients were evaluated for screening. After excluding 101 patients during the screening period, 520 patients were randomly assigned to either the DA-9601 or the misoprostol treatment group. Nine percent of patients in the DA-9601 (24/256) and misoprostol (23/264) groups did not complete the study. Data on the remaining 478 patients were available for the FAS analysis: 236 for DA-9601 versus 242 for misoprostol. Data on 395 patients were available for the PPS analysis: 196 for DA-9601 versus 199 for misoprostol. Figure 1 presents a flowchart of patient progression through the study, with the reasons for premature discontinuation. Baseline characteristics of the patients are presented in Table 2. There were no differences between the groups in terms of gender, age, smoking status and alcohol consumption.

**Table 2:** Demographic and baseline characteristics of study subjects

	DA-9601 (n = 256)	Misoprostol (n = 264)	Total (n = 520)	*P* value
Age(year)^1^	44.1 ± 12.0	44.6 ± 11.9	44.4 ± 11.9	0.6168^a^
Gender				
Male^2^	89 (34.8)	111 (42.1)	200 (38.5)	0.0880^e^
Female^2^	167 (65.2)	153 (58.0)	320 (61.5)	
Height(cm)^1^	163.3 ± 9.1	164.4 ± 9.2	163.9 ± 9.2	0.1622^a^
Weight(kg)^1^	63.9 ± 12.8	63.5 ± 13.2	63.7 ± 13.0	0.7308^a^
Smoking^2^	47 (18.4)	56 (21.2)	103 (19.8)	0.4145^e^
Alcohol^2^	103 (40.2)	106 (40.2)	209 (40.2)	0.9846^e^

^1^Mean ± SD; ^2^ n (%), ^a^ Student’s *t* test, ^e^ Chi squared test

### Efficacy

The FAS population contained 478 patients (236 in the DA-9601 group and 242 in the misoprostol group). The gastric mucosa protection rates were, 81.4 and 89.3 % in the DA-9601 and misoprostol groups at week 4, respectively. The one-sided 97.5 % lower limit was −14.2 %, which is higher than the −17 % margin of non-inferiority, indicating non-inferiority of DA-9601 to misoprostol (Table 3).

**Table 3:** Gastric mucosa protection rate

	DA-9601	Misoprostol
	n (%)	
Full analysis set (FAS)		
	(n = 236)	(n = 242)
Gastric mucosa protection rate	192 (81.4)	216 (89.3)
95 % two-sided exact CI^3^ for protection rate^4^	[75.8, 86.1]	[84.7, 92.9]
97.5 % one-sided lower limit (≥−17) for the difference in protection rate	−14.2	
Per protocol set (PPS)		
	(n = 196)	(n = 199)
Gastric mucosa protection rate	157 (80.1)	179 (89.9)
95 % two-sided exact CI for protection rate	[73.8, 85.5]	[84.9, 93.8]
97.5 % one-sided lower limit (≥−17) for the difference in protection rate	−16.8	

^3^ *CI* confidence interval. ^4^ The exact 95 % CI for protection rate using binomial distribution

In secondary efficacy analyses, the duodenal mucosa protection rate and the ulcer incidence rate were not significantly different between the two groups. The duodenal mucosa protection rates were 98.7 and 98.8 % in the DA-9601 and misoprostol groups, respectively. The 95 % CI for the difference between the groups was −2.0 to 2.0 (Table 4). The ulcer incidence rates were 2.1 and 0.8 % in the DA-9601 and misoprostol groups, respectively. The 95 % CI for the difference between the groups was −0.9 to 3.5 (Table 5).

**Table 4:** Duodenal mucosa protection rate

	DA-9601	Misoprostol	*p* value
	n (%)		
Full analysis set (FAS)			
	(n = 236)	(n = 242)	
Duodenal mucosa protection rate	233 (98.7)	239 (98.8)	1.0000^f^
95 % two-sided exact CI for protection rate	[96.3, 99.7]	[96.4, 99.7]	
95 % two-sided CI for the difference in protection rate	[−2.0, 2.0]		
Per protocol set (PPS)			
	(n = 196)	(n = 199)	
Duodenal mucosa protection rate	194 (99.0)	196 (98.5)	1.0000^f^
95 % two-sided exact CI for protection rate	[96.5, 99.9]	[95.7, 99.7]	
95 % two-sided CI for the difference in protection rate	[−1.7, 2.7]		

^f ^Fisher’s exact test

**Table 5:** Ulcer incidence rate

	DA-9601	Misoprostol	*p* value
	n (%)		
Full analysis set (FAS)			
	(n = 236)	(n = 242)	
Ulcer incidence rate	5 (2.1)	2 (0.8)	0.2799^f^
95 % two-sided exact CI for ulcer development rate	[0.7, 4.9]	[0.1, 3.0]	
95 % two-sided CI for the difference in ulcer development rate	[−0.9, 3.5]		
Per protocol set (PPS)			
	(n = 196)	(n = 199)	
Ulcer incidence rate	5 (2.6)	2 (1.0)	0.2816^f^
95 % two-sided exact CI for protection rate	[0.8, 5.9]	[0.1, 3.9]	
95 % two-sided CI for the difference in protection rate	[−1.1, 4.2]		

^f^Fisher’s exact test

### Safety

The incidence rate of adverse events (AEs), adverse drug reactions (ADRs), severe adverse events (SAEs), and severe adverse drug reactions (SDARs) were not different between the DA-9601 and misoprostol groups (29.9 vs. 31.9 % (*p* = 0.61), 10.4 vs. 14.1 % (*p* = 0.20), 0.4 vs. 0.8 % (*p* = 1.00), and 0 vs. 0.4 % (*p* = 1.00), respectively). GI symptoms were more frequently reported in the misoprostol group than in the DA-9601 group. The mean of the total GI symptom scores for abdominal pain, diarrhea, heartburn, bloating, and anorexia from the baseline to week 4 were lower in the DA-9601 group compared to the mean of the total scores in the misoprostol group. This indicates that the GI symptoms were significantly improved in the DA-9601 group. There was a significant difference between two groups (−0.2 ± 2.8 vs. 1.2 ± 3.2, *p* < 0.0001; Table 6).

**Table 6:** Difference of mean total scores for GI symptoms at week 0 and week 4

	DA-9601		Misoprostol			*p* value
	n, mean ± SD, median, min, max					
Week 0	236	1.4 ± 2.5, 0.0, 0.0, 19.0		242	1.1 ± 2.0, 0.0, 0.0, 9.0	0.1126^b^
Week 4	232	1.2 ± 2.2, 0.0, 0.0, 14.0		240	2.3 ± 3.3, 1.0, 0.0, 22.0	
Difference between week 0 and week 4	232	−0.2 ± 2.8, 0.0, −15.0, 14.0		240	1.2 ± 3.2, 0.0, −6.0, 22.0	<0.0001^b,†^
*P* value		0.1355^d^			<0.0001^d,†^	

^b^Wilcoxon rank-sum test ^d^ Wilcoxon singed-rank test ^†^ *p* < 0.05

## Discussion

The present study evaluated the efficacy of DA-9601 compared with the synthetic prostaglandin E_1_ analogue, misoprostol, in reducing NSAID-associated gastroduodenal complications. This randomized, controlled trial found that the gastric mucosa protection rate in patients on a 4-week NSAID treatment course was not lower when they were administered DA-9601 versus misoprostol. The adverse effect profiles with respect to GI symptoms were improved with DA-9601, compared to misoprostol. These results indicate the non-inferiority of DA-9601 in efficacy and safety, compared with misoprostol.

DA-9601 effectively and safely protected patients from gastric mucosal injury during 4-week NSAID treatment. Our results showed that the gastric mucosa protection rate of the DA-9601 group was high (81.4 %) and was not inferior to that of the misoprostol group (89.3 %). Furthermore, the duodenal mucosa protection rate and the ulcer incidence rate during the 4-week NSAID treatment were not significantly different between the DA-9601 and misoprostol treatment groups. Previously, a similar comparative study in healthy volunteers demonstrated that DA-9601 has a similar efficacy and a favorable safety profile, compared with misoprostol, in the context of protection from NSAID-associated GI complications.

The mechanism of action of DA-9601 in preventing gastric mucosal injury is likely to involve its antioxidant and cytoprotective anti-inflammatory effects. Many studies have demonstrated these effects of DA-9601 on gastric epithelial cells. Eupatilin, a major component of DA-9601, was shown to inhibit the production of FeSO_4_-induced ROS inhibited and reduce oxidative-driven gene expression, resulting in prevention of H_2_O_2_-induced gastric epithelial damage (Choi et al. 2008). In gastric epithelial cells, DA-9601 also inhibited the production of TNF-α, a pro-inflammatory cytokine, through modulation of p38 kinase- and NF-κB-dependent pathways (Choi et al. 2006). Furthermore, DA-9601 has anti-inflammatory and cytoprotective effects in pancreatic and hepatic cells, (Hahm et al. 1998; Ryu et al. 1998). Cytoprotective effects of DA-9601 have been reported by several animal and human studies. DA-9601 significantly improved the histopathology of oxidative injury-induced reflux esophagitis in rats (Oh et al. 2001), and reduced alcohol-induced hemorrhagic injury to the gastric mucosa in rats, by inhibiting alcohol-induced xanthine oxidase (Huh et al. 2003). Moreover, in patients with erosions of the gastric mucosa, DA-9601 effectively and safely healed the gastric mucosa, as assessed by endoscopy (Seol et al. 2004).

Although misoprostol is reported to have greater efficacy than a placebo, and a similar effect to H_2_RAs or PPIs in reducing NSAID-associated gastroduodenal ulcers, low compliance has caused limited use of misoprostol (Weaver and Gitlin 2002; Graham et al. 2002; Hawkey et al. 1998; Raskin et al. 1996; Valentini et al. 1995; McCarthy 2001). Chronic NSAID users may stop taking misoprostol, or physicians may attempt to increase compliance by prescribing a lower dose, resulting in increased risk of NSAID-induced gastroduodenal complications. We found that GI symptoms were significantly lower in the DA-9601 group than in the misoprostol group. As these GI symptoms may be attributable not only to aceclofenac but to misoprostol or DA-9601, it can be speculated that DA-9601 also has a protective effect on NSAID-induced GI symptoms compared with misoprostol.

Regarding the safety of DA-9601, the percentage of patients complaining of any drug-related adverse effects was similar in both groups. AE, ADR, SAE, and SADR were 29.9, 10.4, 1, 0 % in the DA-9601 group, and 31.9, 14.1, 0.8, and 0.4 %, in the misoprostol group, respectively. The difference in the sum of digestive symptoms between before and after the 4-week treatment was significant (−0.2 vs. 1.2 with DA-9601 vs. misoprostol, *p* < 0.0001). Specifically, the symptom scores for diarrhea, heartburn, and bloating between before and after drug treatment were lower in the DA-9601 group than in the misoprostol group. These findings indicate that DA-9601 is more favorable than misoprostol, with respect to drug compliance.

This study assessed NSAID-associated gastroduodenal complications by performing upper GI endoscopy in all participants before and after 4 weeks of treatment. Gastroduodenal lesions visualized by endoscopy were objectively categorized into five grades, according to the severity of mucosal lesions. In those using an NSAID chronically, GI symptoms such as abdominal pain, heart burn, bloating and dyspepsia are frequently noted but these symptoms are unpredictable for gastroduodenal lesions (Larkai et al. 1987). Furthermore, such symptoms are not well correlated with gastroduodenal lesions assessed by endoscopy (Singh and Triadafilopoulos 1999). Although inter-observer variability in assessing gastroduodenal mucosal lesions by endoscopy is possible, the double-blind nature of the study ensured that this method produced data that may be considered objective evidence.

NSAID-induced gastroduodenal complications are known to develop more frequently in patients with risk factors such as steroid or anti-coagulant use, history of a prior peptic ulcer, or advanced age. In the present study, patients with a high risk of NSAID-induced gastroduodenal ulcers were excluded. In these patients, the preferred method of preventing NSAID-associated gastroduodenal complications is to administer a selective COX-2 inhibitor with a PPI, which is a strategy with low cost-benefit ratio (Targownik et al. 2008). The patients enrolled in our study were those requiring chronic NSAID use who were considered to have an average risk of NSAID-induced complications. Therefore, the present study highlights that the cost-benefit ratio of DA-9601 is more acceptable in average-risk patients. Another concern is that one of the important factors for increasing NSAID-induced ulcer is Helicobacter pyloric infection. A meta-analysis concludes that NSAIDs and H. pylori have a synergistic risk for developing peptic ulcer diseases (Huang et al. 2002) and eradication of H. pylori reduces ulcer incidence (Tang et al. 2012). As this factor was not evaluated in the present study participants, this may be another limitation to our study. However, conflicting results also reported that no beneficial effects of H. pylori eradication was obtained for chronic NSAID treatment (de Leest et al. 2007).

In summary, the results of this study indicate that DA-9601 is as effective as misoprostol in protecting against gastroduodenal injury during a 4-week NSAID treatment course. Furthermore, DA-9601 was not inferior to misoprostol in terms of the adverse effect profile. Some of the GI symptoms reported during the study were less frequent in the DA-9601 group than the misoprostol group. Therefore, to prevent NSAID-associated GI complications, we recommend administration of DA-9601 (60 mg, three times per day), rather than misoprostol.
